# Biodiversity of carapace epibiont diatoms in loggerhead sea turtles (*Caretta caretta* Linnaeus 1758) in the Aegean Sea Turkish coast

**DOI:** 10.7717/peerj.9406

**Published:** 2020-07-17

**Authors:** Aydın Kaleli, Ana Car, Andrzej Witkowski, Marta Krzywda, Catherine Riaux-Gobin, Cüneyt Nadir Solak, Yakup Kaska, Izabela Zgłobicka, Tomasz Płociński, Rafał Wróbel, Krzysztof Kurzydłowski

**Affiliations:** 1 Department of Marine and Freshwater Resources Management, Faculty of Aquatic Sciences, Istanbul University, Istanbul, Turkey; 2 Institute for Marine and Coastal Research, University of Dubrovnik, Dubrovnik, Croatia; 3 Institute of Marine and Environmental Sciences, University of Szczecin, Szczecin, Poland; 4 CNRS-EPHE-UPVD, CRIOBE, PSL Research University, Perpignan, France; 5 Laboratoire d’Excellence ‘CORAIL’, Université de Perpignan, Perpignan, France; 6 Department of Biology, Faculty of Science and Arts, Kütahya Dumlupınar University, Kütahya, Turkey; 7 Department of Biology, Faculty of Science and Arts, Pamukkale University, Denizli, Turkey; 8 Faculty of Mechanical Engineering, Bialystok University of Technology, Bialystok, Poland; 9 Faculty of Materials Science and Engineering, Warsaw University of Technology, Warsaw, Poland; 10 Faculty of Chemical Technology and Engineering, West Pomeranian University of Technology, Szczecin, Poland

**Keywords:** Diatoms (Bacillariophyta), Biodiversity, *Caretta caretta*, Epibionts, The Mediterranean Sea, Turkey

## Abstract

**Background:**

The Aegean Sea coast of Turkey hosts one of the most important nesting grounds for loggerhead sea turtles (*Caretta caretta*) in the Mediterranean Sea. Previous studies have revealed that the sea turtle carapace provides favourable conditions for various epibiontic organisms. Epibionts occurring on the carapace have been examined from different locations in the oceans.

**Methods:**

This is the first time such a high number (39) of samples collected from nesting turtles during such a long time period (extending from 2011 to 2018) has been used for the study of the diatom component of the microbiome on the turtle carapaces. A total of 33 samples were investigated in terms of light microscopy (LM) and scanning electron microscopy (SEM). Six unprocessed biofilm fragments were subject to SEM observations.

**Results:**

A total of 457 epizoic diatom taxa belonging to 86 genera were identified. Epizoic forms, e.g., *Achnanthes* spp., *Chelonicola* spp. or *Tripterion* spp. (also identified by SEM observations of the undisturbed pieces of the microbiome) dominated in terms of relative abundance, but the highest numbers of taxa were ubiquitously represented by *Navicula* (79), *Nitzschia* (45), *Amphora* (40), *Cocconeis* (32), *Diploneis* (25) and *Mastogloia* (23). *Navicula perminuta* and *Delphineis australis* were the most frequent taxa, present in 65% of the samples, both with an average relative abundance of 10%. The results of our study revealed that diatoms are an essential component of the loggerhead sea turtles’ microbiome, in terms of high biodiversity and abundance. Although strict epibionts provide a signature of the turtle microbiome, the carapace as a solid substrate attracts numerous benthic diatom species which are considered opportunistic forms and can be found in the surrounding benthic habitats of the vast ocean littoral space.

## Introduction

Epibiosis is a relationship between two organisms where an epibiont lives on the surface of a basibiont used as a substrate (Lima et al., 2017). Marine vertebrates (especially whales and sea turtles) are ideal motile substrata for other organisms and are known to host epibiont assemblages (Dodd, 1988; Ernst, Barbour & Lovich, 1994). Although there has been much focus on the epibiont fauna of sea turtles, scientists have begun also to investigate the epibiont flora of sea turtles in recent decades. Kitsos et al. (2005) found seventeen taxa of algae associated with loggerhead sea turtles from Greek coasts. Green and red-algal taxa have been found on sea turtles (Pfaller et al., 2006; Pfaller et al., 2008), including a newly described Rhodophyte species limited in its distribution to turtles inhabiting the Mediterranean Sea (Báez et al., 2001).

Although epizoic diatoms on vertebrates were first described from cetaceans, freshwater and sea turtles can also host very specific diatom floras (Nemoto, 1956; Holmes, Nagasawa & Takano, 1993a; Holmes, Nagasawa & Takano, 1993b; Denys, 1997; Riaux-Gobin et al., 2017a; Riaux-Gobin et al., 2017b). Loggerhead sea turtles (*Caretta caretta* Linnaeus, 1758) are one of the seven species of sea turtles (Lutz & Musick, 1997), distributed from tropical waters of the Indian and the Pacific Ocean to temperate waters of the Atlantic Ocean and the Mediterranean Sea (Ernst, Barbour & Lovich, 1994). The most recent research on epibionts from extant sea turtle microbiomes showed that diatoms are present on all known species of turtles (Robinson et al., 2016). The same authors found that the sea turtle carapace could be host to several undescribed taxa (Robinson et al., 2016). There have been a number of recent papers with analyses of the epibiont diatom composition on the carapace of the sea turtles (Frankovich, Sullivan & Stacy, 2015; Majewska et al., 2015a; Majewska et al., 2015b; Majewska et al., 2017a; Riaux-Gobin et al., 2017a; Riaux-Gobin et al., 2017b). Several diatom genera and species have been described as new to science from the carapace of sea turtles from different geographic regions. Majewska et al. (2015a) described two genera (*Poulinea* Majewska, De Stefano & Van de Vijver and *Chelonicola* Majewska, De Stefano & Van de Vijver) from olive ridley sea turtles (*Lepidochelys olivacea* Escholtz, 1829) from the Pacific coast of Costa Rica. *Chelonicola caribeana* Riaux-Gobin, Witkowski, Ector & Chevallier and *Tripterion societatis* Riaux-Gobin, Witkowski & Ector were identified and described from the Atlantic Ocean from green sea turtle (*Chelonia mydas* Linnaeus, 1758) population (Riaux-Gobin et al., 2017b). Additionally, *Tursiocola yin-yangii* Riaux-Gobin & Witkowski and *Tursiocola guyanensis* Riaux-Gobin & Witkowski were described from green turtles in French Guiana and the eastern Caribbean (Riaux-Gobin et al., 2017a). Research on *Tursiocola* and *Tripterion* species revealed that some epibiont diatoms could live on various animals’ skin or carapaces. In the past *Tursiocola* species have been observed on Dall’s porpoises (*Phocoenoides dalli* True, 1885) (Nemoto, 1956, Holmes, Nagasawa & Takano, 1993a; Holmes, Nagasawa & Takano, 1993b; Denys, 1997), on manatee (*Trichechus manatus* Linnaeus, 1758) skin (Frankovich, Sullivan & Stacy, 2015) and freshwater turtles (Wetzel et al., 2012). Some *Tripterion* species were formerly reported from whales and other cetaceans.

In the Mediterranean Sea, the most numerous turtle nesting sites are on the northern Cilician coasts of Turkey. Recently, diatoms associated with the Mediterranean loggerhead sea turtle population have been described. These included an *Olifantiella* species (Kaleli et al., 2018) and six new species of *Proschkinia* (Majewska et al., 2019), and a small celled *Catenula* taxon from the Adriatic Sea (Robert, Bosak & Van de Vijver, 2019).

The objectives of this study were (i) to describe the species composition and diversity of diatom assemblages on loggerhead sea turtles from a series of survey samples taken between 2011–2014, (ii) to determine functional group of particular diatom taxa e.g., epizoic, epiphytic and (iii) to highlight data on the diatom species associated with the biofilm from the samples collected in 2018 which have been studied in situ with SEM.

## Material & Methods

### Study area

**Figure 1:**
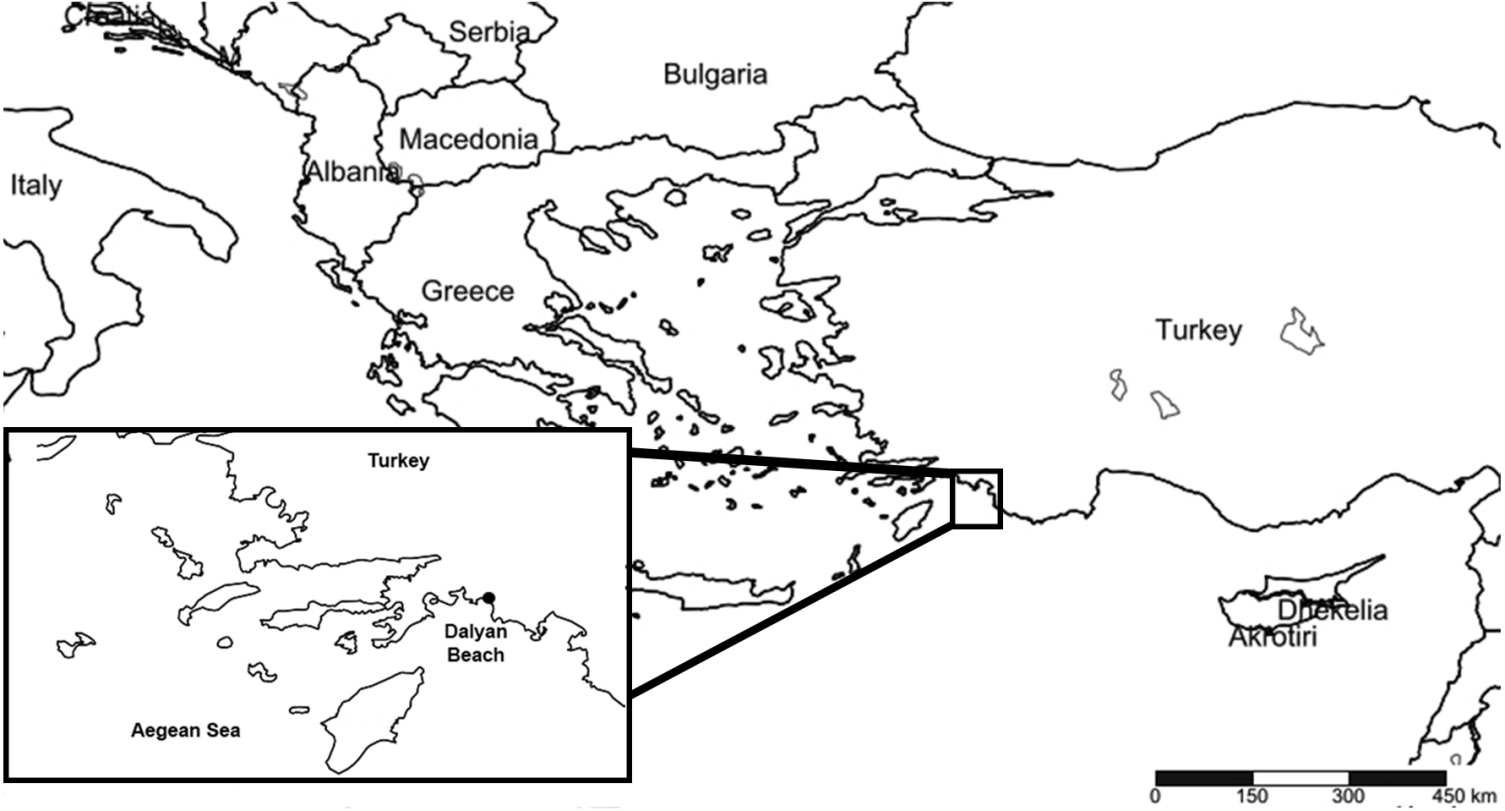
Location of the sampling site.

Dalyan beach is located in the province of Muğla (36°42′02′′N, 28°41′31′′E) (Fig. 1). It has one of the highest numbers of loggerhead sea turtle nests along with the beaches of Belek, Antalya, and Anamur, along the Aegean and the Mediterranean coasts of Turkey (Kaska et al., 2016). As a result, Dalyan beach was assigned as a “specially protected area” in 1988 and has “flagship beach” status for the conservation of loggerhead sea turtles (Türkozan & Yılmaz, 2008). The beach is 4.7 km long and composed of a fine-sand dune and gravel drifted from the Dalyan Delta, which is deposited to the east of the beach. Dalyan Delta is an extensive wetland with a labyrinth of reedy channels opening to Köyceğiz Lake via the Dalyan River where, during the study period (2011–2018), some foraging sea turtles were observed. The wetland complex (Dalyan Delta) opens to the sea through a channel at the northern part of the beach (Türkozan & Yılmaz, 2008).

### Sampling

**Figure 2:**
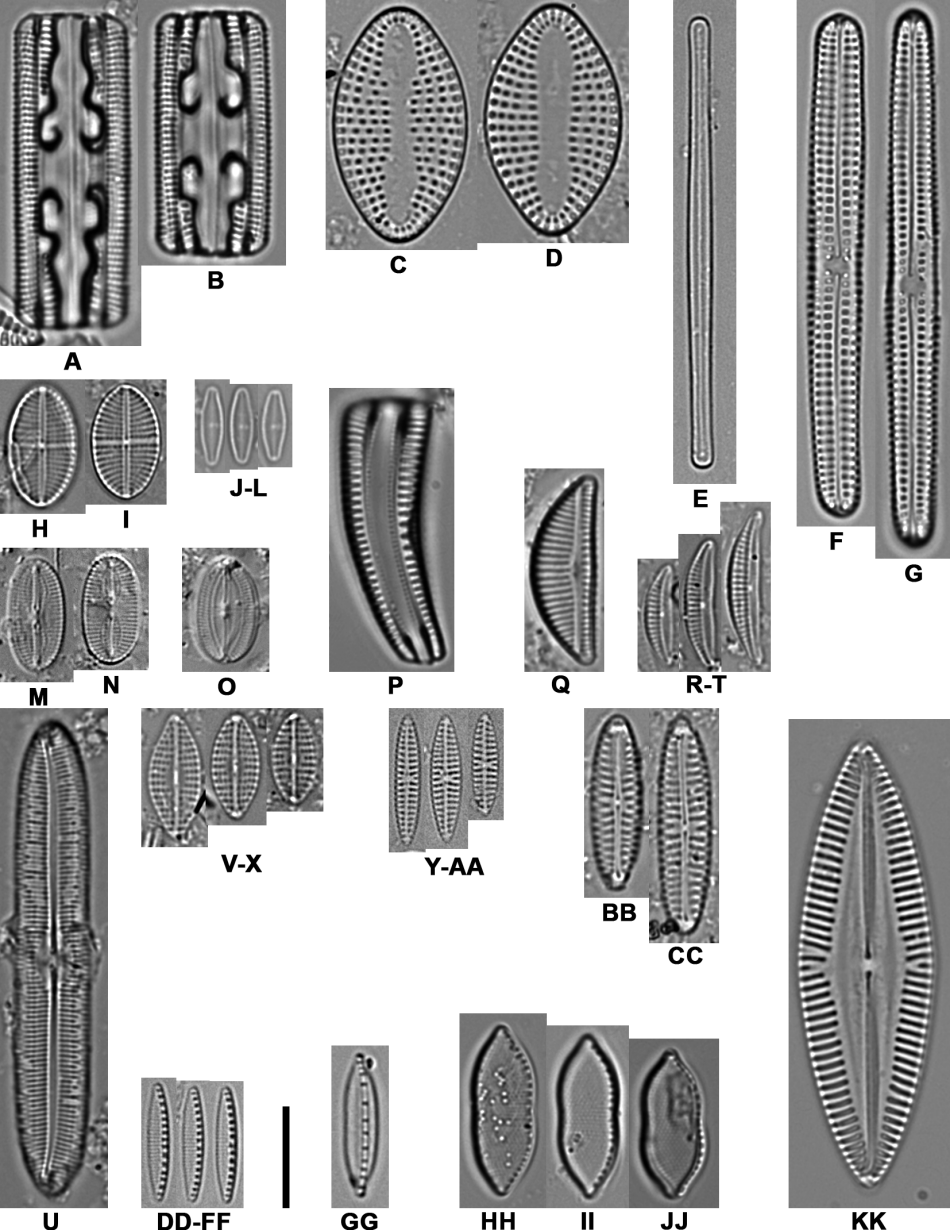
Light microscope images of the most abundant epibiont diatoms associated with *Caretta caretta*. (A, B) *Grammatophora angulosa*; (C, D) *Delphineis australis*;** (E) *Neosynedra provincialis*; (F, G) *Achnanthes elongata*; (H, I) *Mastogloia crucicula* var. *alternans*; (J–L) * Olifantiella seblae*; (M, N) *Fallacia cassubiae*; (O) *Fallacia florinae*; (P) *Rhoicosphenia abbreviata*; (Q) *Encyonema minutum*; (R–T) *Halamphora tenerrima*; (U) *Caloneis liber*; (V–X) *Navicula vimineoides*; (Y–AA) *Navicula perminuta*; (BB, CC) *N.* cf. *borowkae*; (DD–FF) *Nitzschia frustulum*; (GG) *N. volvendirostrata*; (HH–JJ) *Psammodictyon rudum*; (KK) *N. palpebralis* var. *angulosa*. Scale bar: 10 µm.

Samples of diatoms were collected from nesting loggerhead sea turtles, at night during the nesting season, between May–August, 2011–2014 and 2018 (Fig. 2). All sampling was carried out in accordance with the regulations of the Ministry of Environment and Urbanization (TR-15/04/2018/39). Sampling was supervised by experts from the Sea Turtle Research Rescue and Rehabilitation Centre (DEKAMER), Ref. B.32.PAU.0.AG.00.00/005. In total, 39 samples were taken. Samples were collected with toothbrushes from 20 cm^2^ of vertebral and coastal carapace scutes of 33 turtles (curved carapace length (CCL) between 67,5–77 cm) between 2011–2014, and pieces of biofilm were scraped with a razor from six different sea turtles (according to the conservation regulations) while the turtles were laying eggs in 2018. A total of 33 samples were processed and used for light microscopy (LM) and scanning electron microscopy (SEM) (3 samples from 2011; 5 samples from 2012; 20 samples from 2013 and 5 samples from 2014). Six unprocessed fragments of biofilm (from 2014 and 2018) were used for SEM observations (Table 1).

**Table 1:** Sampling codes of the carapaces. Note that CAR_2018_5 was taken from a dead sea turtle carapace, CAR_2013_10 is the cleaned material, and CAR_2018_4 is the biofilm fragment from the same turtle that was sampled in 2013 and 2018.

**Fieldwork—culture collection code names**	**Codes names adjusted for this study**	**Sampling date**
18866 / TRYB-404	CAR_2011_1	**2011**
18867 / TRY-0074	CAR_2011_2	2011
18868 / TRY-0075	CAR_2011_3	2011
19772	CAR_2012_1	**2012**
19776	CAR_2012_2	2012
19780	CAR_2012_3	2012
19781	CAR_2012_4	2012
19782	CAR_2012_5	2012
20679 / TRY-0200	CAR_2013_1	**2013**
20690 / TRY-0008	CAR_2013_2	2013
20694 / TRY-0141	CAR_2013_3	2013
20698 / TRY-0412	CAR_2013_4	2013
20705 / TRY-0027	CAR_2013_5	2013
20707 / TRY-0175	CAR_2013_6	2013
20714 / TRY-0130	CAR_2013_7	2013
20715 / TRY-0138	CAR_2013_8	2013
20735 / TRY-0174	CAR_2013_9	2013
TRC-2300	CAR_2013_10	2013
TRY-0154	CAR_2013_11	2013
TRY-0165	CAR_2013_12	2013
TRY-0184	CAR_2013_13	2013
TRY-0438	CAR_2013_14	2013
TRY-0439	CAR_2013_15	2013
TRY-0442	CAR_2013_16	2013
TRY-0451	CAR_2013_17	2013
TRY-0452	CAR_2013_18	2013
TRY-0457	CAR_2013_19	2013
TRY-0467	CAR_2013_20	2013
Caretta 2014-1	CAR_2014_1	**2014**
Caretta 2014-2	CAR_2014_2	2014
Caretta 2014-3	CAR_2014_3	2014
Caretta 2014-4	CAR_2014_4	2014
Caretta 2014-6	CAR_2014_5	2014
**Biofilm fragments**		
TRY-0520	CAR_2014_6	**2014**
TRY-0627	CAR_2018_1	**2018**
TRY-1180	CAR_2018_2	2018
TRY-2012	CAR_2018_3	2018
TRC-2300	CAR_2018_4	2018
TRY-Carapace-1801	CAR_2018_5	2018

Biofilm pieces were fixed with 70% ethanol for 4 h. Each fixed biofilm was then washed five times with distilled water, followed by washing in increasing alcohol concentration. In each concentration, the biofilm was left for 20 mins, (30 mins in absolute alcohol) at room temperature. After drying, a piece of biofilm was mounted on an aluminium stub with double-adhesive carbon tape. Untreated samples of the dried and dehydrated microbiome were sputter-coated with palladium-gold alloy and observed with a Hitachi SU8020 scanning electron microscope (Hitachi, Tokyo, Japan).

For light (LM) and scanning electron microscopy (SEM) observations, samples were cleaned to remove organic material by washing with 10% HCl, boiling in 30% H_2_O_2_ and rinsing with distilled water (Swift, 1967). Permanent slides were air-dried and mounted in Naphrax^®^. LM observations were performed with a Zeiss Axio Imager 2 (Carl Zeiss Microscopy Gmbh, Jena, Germany) equipped with a 100 × oil immersion Plan apochromatic objective (with numerical aperture = 1.46) at the University of Szczecin (Poland), and a Nikon Eclipse Ci (Nikon Corp. Tokyo, Japan) with a Nikon DS-Fi1 camera at the Kütahya Dumlupınar University. SEM images were taken using a HITACHI S-5500 at Warsaw University of Technology (Poland). Slides and processed material are deposited at the Department of Marine and Freshwater Resources Management, Istanbul University, Istanbul (Turkey) and the diatom collection (SZCZ) of the Institute of Marine and Environmental Sciences, University of Szczecin, Szczecin (Poland).

### Data analysis

The abundance of diatom species was expressed as a percentage of the total number of valves counted (relative abundances in %). The relative abundance (RA) of particular taxa and the taxa richness of the assemblages were estimated on the basis of at least 300 diatom valves counted per sample. Frequency of the most abundant taxa and their maximum RA during the four-year period (2011–2014) and for each of the years were determined.

Raw diatom counts were expressed as a relative abundance and were square-root transformed to normalize data. A resemblance matrix of the data was generated using Bray–Curtis analysis. The Bray–Curtis similarity matrix (Legendre & Legendre, 1983; Clarke & Gorley, 2006) of the relative abundance data of 457 taxa over 33 samples was constructed. Similarity percentage analysis (SIMPER, (Clarke & Warwick, 1994)) was used to identify the taxa making the most significant contribution to the similarities between epibiontic diatom assemblages. All statistical analyses were performed using the Primer v6 software (Clarke & Gorley, 2006) and Statistica 7.0 (StatSoft, Inc. 2004).

Identifications were made following Witkowski, Lange-Bertalot & Metzeltin (2000). Terminology follows Round, Crawford & Mann (1990), and nomenclature of recorded taxa follows AlgaeBase (Guiry & Guiry, 2019) and Diatombase (Kociolek et al., 2019).

## Results

### Diatom composition & distribution

A total of 457 diatom taxa belonging to 86 diatom genera were identified from 33 samples (Table S1). Among them, 62, 95, 253 and 275 taxa were identified in 2011, 2012, 2013 and 2014, respectively. Among the 457 diatom taxa, 27 taxa were observed exclusively in 2011, 26 taxa in 2012, 111 taxa in 2013, and 129 taxa in 2014, while 174 taxa were found only once (sporadic).

The genera with the highest number of taxa represented were *Navicula* (79), *Nitzschia* (45), *Amphora* (40), *Cocconeis* (32), *Diploneis* (25), *Mastogloia* (23), *Fallacia* (14) and *Achnanthes* (12), followed by *Halamphora* (10) and *Psammodictyon* (10). Although *Navicula* and *Nitzschia* had the highest numbers of taxa, they occurred with an average RA of 3%. Amongst the genera which were recorded in all four sampling years, the most abundant was *Achnanthes* (Avg RA = 7%) (Tables 2 and 3).

**Table 2:** Dominant diatom taxa collected during a four-year period (2011–2014) from turtles with the frequency of appearance (Freq.) >3%, average relative abundance (Avg. RA) >6% and maximum relative abundance (Max. RA) >6%. *N* = 33.

**Taxa**	**Freq.** **(%)**	Avg. RA **(%)**	Max. RA **(%)**	Sampling year of Max. RA
*Achnanthes elongata* Majewska & Van de Vijver	35.29	19.43	65.71	2013
*Caloneis liber* (Smith) Cleve	2.94	6.67	6.67	2013
*Delphineis australis* (Petit) Watanabe et al.	64.71	9.62	33.33	2013
*Dickieia* sp.1	2.94	18.37	18.37	2011
*Encyonema minutum* (Hilse) D.G.Mann	8.82	7.07	16.67	2013
*Fallacia cassubiae* Witkowski	2.94	10.20	10.20	2011
*Fallacia florinae* (Møller) Witkowski	2.94	8.16	8.16	2011
*Grammatophora angulosa* Ehrenberg	55.88	6.63	50.00	2013
*Halamphora tenerrima* (Aleem & Hustedt) Levkov	32.35	5.99	25.85	2011
*Mastogloia crucicula* var. *alternans* Zanon	11.76	12.69	50.00	2013
*Navicula* cf. *borowkae* Witkowski et al.	2.94	12.93	12.93	2011
*Navicula palpebralis* var. *angulosa* (Gregory) Van Heurck	2.94	6.67	6.67	2013
*Navicula perminuta* Grunow	64.71	9.84	75.00	2013
*Navicula* sp. 13	5.88	12.56	25.00	2013
*Navicula vimineoides* Giffen	2.94	18.59	18.59	2011
*Neosynedra provincialis* (Grunow) Williams & Round	5.88	10.40	20.00	2013
*Neosynedra* sp. 1	5.88	12.08	20.00	2013
*Nitzschia frustulum* (Kützing) Grunow	55.88	14.63	58.02	2012
*Nitzschia volvendirostrata* Ashworth et al.	2.94	50.00	50.00	2013
*Olifantiella seblae* Kaleli et al.	5.88	12.41	24.00	2012
*Parlibellus* sp. 1	2.94	6.12	6.12	2011
*Psammodictyon rudum* (Cholnoky) Mann	29.41	7.47	60.00	2013
*Rhoicosphenia abbreviata* (Agardh) Lange-Bertalot	2.94	50.00	50.00	2013
*Tripterion* sp. 2	35.29	9.48	37.50	2013

**Table 3:** List of diatom taxa and their percentage contribution to total diatom community composition (taxa with relative abundances, RA ≥ 5% are only shown) from 2011 till 2014.

**Year**	**2011**		**2012**		**2013**		**2014**	
****		**RA (%)**	****	**RA (%)**	****	**RA (%)**	****	**RA (%)**
**Taxa**	*Karayevia submarina* (Hustedt) Bukhtiyarova	33.33	*Achnanthes elongata* Majewska & Van de Vijver	60.61	*Achnanthes elongata* Majewska & Van de Vijver	27.57	*Nitzschia frustulum* (Kützing) Grunow	13.05
	*Navicula vimineoides* Giffen	18.59	*Nitzschia frustulum* (Kützing) Grunow	31.10	*Mastogloia crucicula* var. *alternans* Zanon	25.05	*Navicula perminuta* Grunow	9.60
	*Dickieia* sp.1	18.37	*Olifantiella seblae* Kaleli et al.	12.41	*Navicula* sp.13	25.00	*Delphineis australis* (Petit) Watanabe et al.	6.11
	*Halamphora tenerrima* (Aleem & Hustedt) Levkov	14.44	*Tripterion* sp.2	9.10	*Tripterion* sp.2	18.06	*Tabularia fasciculata* (Agardh) Williams & Round	5.95
	*Navicula* cf. *borowkae* Witkowski et al.	12.93	*Halamphora tenerrima* (Aleem & Hustedt) Levkov	7.97	*Delphineis australis* (Petit) Watanabe et al.	12.40	*Cocconeis placentula* Ehrenberg	5.28
	*Navicula perminuta* Grunow	12.12	*Navicula perminuta* Grunow	7.92	*Brachysira estonarium* Witkowski et al.	12.16		
	*Fallacia cassubiae* Witkowski	10.20	*Halamphora luciae* (Cholnoky) Levkov	7.59	*Neosynedra* sp.1	12.08		
	*Fallacia* sp.1	8.39			*Psammodictyon rudum* (Cholnoky) Mann	11.74		
	*Fallacia florinae* (Møller) Witkowski	8.16			*Neosynedra provincialis* (Grunow) Williams & Round	10.40		
	*Cocconeis latecostata* Hustedt	8.16			*Navicula perminuta* Grunow	10.39		
	*Parlibellus* sp.1	6.12			*Nitzschia frustulum* (Kützing) Grunow	8.84		
	*Hippodonta* sp.1	6.06			*Grammatophora angulosa* Ehrenberg	8.60		
	*Fallacia oculiformis* (Hustedt) Mann	5.78			*Halamphora luciae* (Cholnoky) Levkov	7.89		
	*Fallacia subforcipata* (Hustedt) Mann	5.33			*Encyonema minutum* (Hilse) D.G.Mann	7.07		
					*Planothidium lilljeborgei* (Grunow) Witkowski et al.	7.02		
					*Navicula palpebralis* var. *angulosa* (Gregory) Van Heurck	6.67		
					*Caloneis liber* (Smith) Cleve	6.67		
					*Brachysira aponina* Kützing	6.46		
					*Navicula normaloides* Cholnoky	5.92		
					*Tryblionella granulata* (Grunow) Mann	5.78		
					*Nitzschia liebetruthii* Rabenhorst	5.23		
					*Achnanthes brevipes* Agardh	5.12		

**Figure 3:**
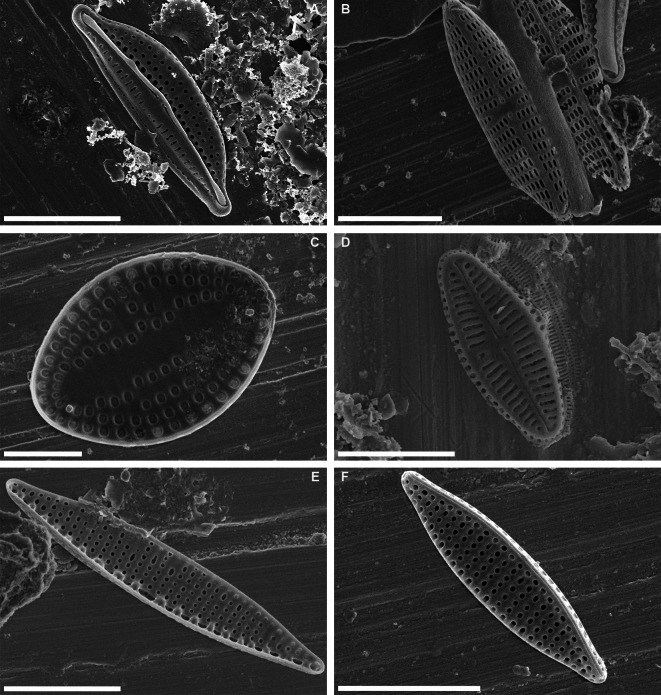
Scanning electron micrographs of some abundant taxa in epibiont diatom assemblages associated with *Caretta caretta*. (A) *Halamphora tenerrima*; (B) *Navicula perminuta*; (C) *Delphineis australis*; (D) *Olifantiella seblae*; (E, F) *Nitzschia frustulum*. Scale bars: (A, B, C, E, F): 5 µm; (D): 3 µm.

The results revealed that there were 16 taxa common to all four sampling years. These taxa were *Achnanthes elongata* Majewska & Van de Vijver, *Cocconeis* sp. 8, *Dimmeregramma minus* var. *nanum* (Gregory) Van Heurck, *Diplomenora cocconeiformis* (Schmidt) Blazé, *Diploneis bombus* (Ehrenberg) Ehrenberg, *Halamphora acutiuscula* (Kützing) Levkov, *H. tenerrima* (Aleem & Hustedt) Levkov, *Karayevia submarina* (Hustedt) Bukhtiyarova, *Meloneis mimallis* Louvrou, Danielidis & Economou-Amilli, *Navicula normaloides* Cholnoky, *N. perminuta* Grunow, *Nitzschia elegantula* Grunow, *N. liebetruthii* Rabenhorst, *Pinnunavis yarrensis* (Grunow) Okuno, *Tryblionella pararostrata* (Lange-Bertalot) Lange-Bertalot, *T. granulata* (Grunow) Mann. *Navicula perminuta* and *Delphineis australis* (Petit) Watanabe, Tanaka, Reid, Kumada & Nagumo were recorded in 65% of samples, both with an average RA of 10% (Figs. 2 and 3, Tables S2–S5).

According to the SIMPER analysis (Tables S2–S5), samples collected from turtles in 2014 had the highest observed within-group average similarities (37.96%). As revealed by SIMPER analyses, the group of taxa contributing the most (cumulatively 50.63%) to similarity between diatom assemblages from the five samples collected in 2014 included *Navicula perminuta*, *Nitzschia frustulum*, *Cocconeis placentula* Ehrenberg, *Navicula* sp. 54, *Navicula* sp. 55, *Nitzschia liebetruthii*, *Melosira moniliformis* (Müller) Agardh, *Tryblionella granulata* and *Seminavis strigosa* (Hustedt) Danielidis & Economou-Amilli (Table S5).

### Biofilm observations

**Figure 4:**
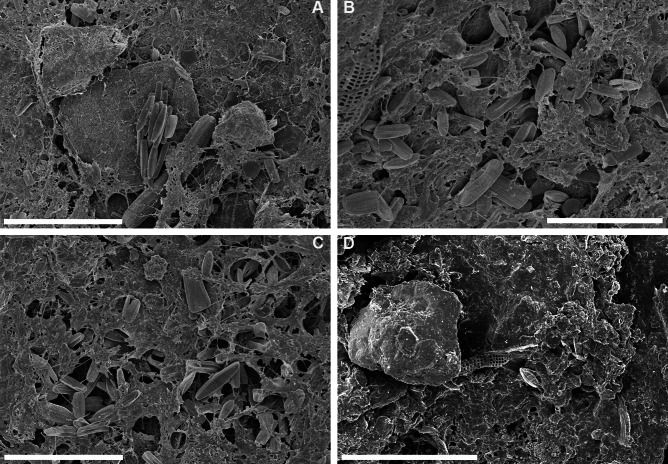
SEM observations of intact biofilm fragments from *Caretta caretta*. (A) aggregate of *Navicula* sp. with *Achnanthes* sp. and solitary valves of epizoic diatoms between mucilage and pieces of the carapace. (B, C) same biofilm rich in epizoic diatoms mainly *Tripterion* sp. with solitary specimens of *Navicula* and *Nitzschia* spp. intercalated with mucilage. (D) Diatom poor example of biofilm with rare fragmented diatoms; note the presence of mineral detritus. ((A–C) CAR_2018_3; D. CAR_2018_2). Scale bars: (A): 50 µm; (B): 20 µm; (C): 30 µm; (D): 40 µm.

**Figure 5:**
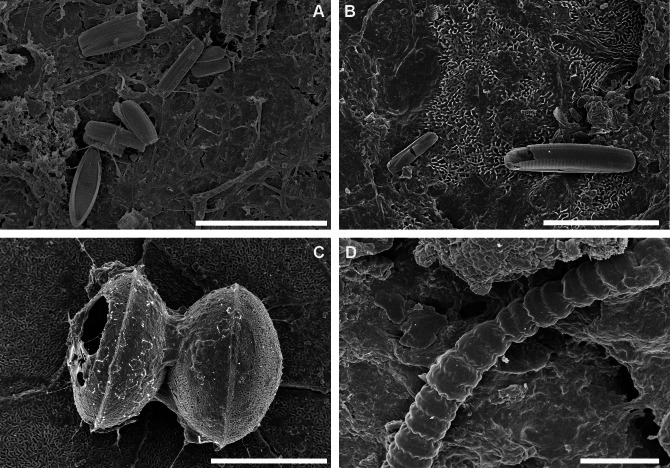
SEM observations of intact biofilm fragments from *Caretta caretta*. (A) Biofilm rich in epizoic diatoms mainly *Tripterion* sp. with solitary specimens of *Navicula* and *Nitzschia* spp. intercalated with mucilage. (B) Exposed surface of carapace with rare broken diatom frustules and mucilage. (C) Chain-forming *Melosira* sp. attached with mucilage to the carapace surface. (D) Close up of filamentous Cyanobacteria - *Anabaena* sp. Note the presence of mucilage and absence of diatoms. ((A) CAR_2018_2; (B) CAR_2018_5; (C) CAR_2018_1; (D) CAR_2018_4 samples). Scale bars: (A, B, C): 20 µm; (D): 10 µm.

During SEM analysis of the unprocessed biofilm samples (Figs. 4, 5) diatoms were found mixed with other microorganisms, e.g., cyanobacteria, organic detritus, broken pieces of the carapace, mineral detritus and diatomaceous detritus. However, in the carapace fragments (CAR_2018_1 and CAR_2018_5), which had sparse biofilm components, diatoms were observed as pioneer epibionts attached directly to the carapace. In the well-developed biofilm (CAR-2018_3) diatoms were abundant, well preserved and represented by epizoic forms: *Achnanthes elongata*, *A*. *squaliformis* (Majewska et al., 2017a), *Chelonicola* sp. and *Tripterion* spp. Another biofilm was dominated by cosmopolitan species such as *Navicula perminuta* and small *Nitzschiae* sect. Lanceolatae (*N*. f*rustulum*, *N*. *liebethrutii*), with lesser participation of the above-mentioned epizoic forms. It appeared as if the layers of diatoms were bound between microlayers of a mucilage composed of unidentifiable organic matter, possibly containing microfungi. In the well-developed biofilm fragments, low occurrence of diatoms was observed. Biofilm sample CAR_2018_2 (Fig. 4D) was mostly composed of mineral and fine organic detritus along with relatively rare, usually broken, diatom frustules. Interestingly, in CAR_2018_4 (Fig. 5D), we observed organic compounds, filamentous cyanobacteria (*Anabaena* sp.) and fine-mineral detritus, whereas diatoms were absent. The differences on the biofilm of several loggerhead sea turtles may give ideas of the development of biofilms, also diatom composition should be taken into consideration. However there are not any data on sea turtles’ health regarding diatoms, diatom composition especially freshwater and brackish taxa may be monitored in foraging areas. Komoroske et al. (2011) observed concentrations of pollutants of carapace like metals, in further studies diatom composition and pollutants could be monitored to reveal any possible relation.

## Discussion

### Diatom composition

In this study, we present the first detailed floristic list of epibiont diatoms observed on the carapace of loggerhead sea turtles in the Mediterranean Sea. The number of taxa (457) was higher than any floristic surveys conducted on turtles or similar biotic habitats (e. g., whales and cetaceans) in the Mediterranean and over a wider geographic area (Nemoto, 1956; Nemoto, 1958; Majewska et al., 2015b; Robinson et al., 2016). The number of diatom taxa recorded in this study was considerably larger than those recorded on the carapace of olive ridley sea turtles (21 diatom taxa) in Majewska et al. (2015b) and green sea turtles carapace (57 diatom taxa) in Rivera et al. (2018). Number of diatom taxa difference might be related with some factors such as sample numbers, sampling techniques (razoring or brushing the carapce), sea turtles’ foraging areas (Robinson et al., 2016) or the possible difficulties in marine diatom identification. However common diatom taxa of the three sea turtle species might suggest that not only the epizoic diatoms addressed to sea turtles but also other marine diatoms could occur on different sea turtle species, whereas further studies on sea turtle species may reveal the similarity or difference on diatom dispersal.

Of the 457 diatom taxa, the genus *Navicula* was the most diversified, with several unidentified *Navicula* spp. abundant in the samples analysed. However, most of the unidentified *Navicula* spp. were similar in morphology with only very minor differences in some characteristics. This might be a result of adaptation in the biofilm (e.g., some valves were heteropolar with a narrower valve end on one side of the valve). The second-largest group in the diatom community was *Nitzschia* spp., with *N. frustulum* as the dominant taxon overall. We observed some small-celled taxa, e.g., *Nitzschia inconspicua*, *Halamphora tenerrima* and *Navicula* spp. on the turtle carapaces, in accordance with previous studies from various regions (Majewska et al., 2015b; Robinson et al., 2016; Rivera et al., 2018). Occurrence on the turtle carapace might be related to the small cell size of the frustules, which may lead to rapid reproduction, as has been observed in *Navicula perminuta* (an opportunistic species). *Mastogloia* species were found in low abundance, but were represented by numerous species (e.g., *M*. *adriatica* Voigt, *M*. *corsicana* Grunow, and *M*. *decussata* Grunow). Therefore, *Mastogloia* species demonstrated the ability to survive under conditions in the biofilm, but were unable to reach high abundance.

### Comparison with the local diatom flora

Some of the taxa observed in the biofilm on the loggerhead sea turtle carapace have been found in diatom flora on different substrata in the same region and along the Aegean Sea coast, and do not seem to have a preference either for a geographic region or for the substrate type (Kaleli, 2019; Kaleli, Kociolek & Solak, 2020). In a shallow coastal lake (Iztuzu Lake), in the same area as the beach occupied by sea turtles during the nesting season, diatoms were abundant (Kaleli, 2019), and some of the species were the same as those found associated with the *C. caretta* carapace (e.g., *Diplomenora cocconeiformis*, *Diploneis bombus*, *Fallacia schaeferae* (Hustedt) D.G. Mann, *Mastogloia lanceolata*, *Meloneis mimallis*). It is possible that diatoms were transferred by the sea turtles during the nesting season. These taxa have also been observed from different locations in the adjacent coasts and also in the Western Indian Ocean (Kaleli et al., 2018, unpublished observations).

Despite the fact that marine taxa strongly dominated the assemblages (Table S3), a few freshwater taxa were observed. The freshwater forms were usually observed as solitary valves (e.g., *Encyonema minutum* (Hilse) D.G. Mann and *Lindavia balatonis* (Pantocsek) Nakov, Guillory, Julius, Theriot & Alverson). The presence of taxa associated with fresh to brackish waters (Table S6) was not particularly surprising as Köyceğiz Lake, which is a typical freshwater lake, is located nearby, and connected through the delta, to Dalyan beach. Both male and female turtles have been observed in the shallow waters close to the banks of the channels connecting the beach to Köyceğiz Lake. Some of the turtles were also observed feeding in the lake and this could be why freshwater taxa were incorporated into the biofilm. Some taxa may be able to tolorate change in salinity (freshwater-brackish, brackish-marine) despite their apparent freshwater preference, and results also support the idea that some species could have different responses to environmental conditions, resulting in a better or worse adaptation (Underwood, Phillips & Saunders, 1998; Ribeiro et al., 2003; Miho & Witkowski, 2005; Hafner, Jasprica & Car, 2018) to variable conditions, which could be explained by the number of the freshwater forms observed on the carapace. It was also suggested by Majewska et al. (2017b) that lakes and rivers could make exclusive epibiosis where specific species could attach and grow in the biofilm and environment affects the dispersal on sea turtle carapace. The abundance of freshwater and brackish water species, presumably reveal that important amount of sea turtles access to shallow freshwaters of Dalyan and spend long periods in the surrounding areas. Nutrient enrichment in these waters provide favourable conditions for the ubiquitous taxa. *Navicula perminuta* and *Nitzschia frustulum*, which are found in marine and brackish waters, dominated the assemblages and this may indicate that species with similar ecological tolerances can settle on the carapace, but species with better adaptation (small cell size, attaching to the carapace, broad tolerance of changes in salinity and light intensity) can thrive.

### Epizoic diatoms

Among the dominant taxa, *Achnanthes elongata* and *Olifantiella seblae* (Kaleli et al., 2018), recently described from the same biofilm samples, were observed, as obligately epizoic diatoms together with unidentified *Chelonicola* sp. and *Tripterion* spp. which we consider potentially new to science (Kaleli et al. *in preparation*). Representatives of *Chelonicola* and *Tripterion* have been described and observed as obligately epizoic forms on sea-turtle carapaces from oceanic waters (Majewska et al., 2015a; Riaux-Gobin et al., 2017a; Riaux-Gobin et al., 2017b) and have not yet been found on other substrates. *Achnanthes elongata* was described from samples from the Pacific coasts and with this study observed for the first time in the Mediterranean Sea, *O*. *seblae* has only been observed in the Mediterranean Sea (Kaleli et al., 2018). The epizoic taxa observed in this study (*O*. *seblae*, *A*. *elongata*) have a broad range of valve morphology in terms of outline. Morphological plasticity is common in diatom species observed on other sea turtle carapaces or whale skin (Nemoto, 1956; Nemoto, 1958; Riaux-Gobin et al., 2019). For example, *Olifantiella* showed high plasticity in the Mediterranean samples, and *Olifantiella seblae* was observed with a length range of 4.5–14.5 µm with elliptic–lanceolate valves. A recent study on *Olifantiella* species from the South Pacific found similar results on valve plasticity (Riaux-Gobin et al., 2019), valve outline had a wide range of polymorphisms and changes were observed also in valve structure, such as stria formation and counts and the buciniportula, though it was indicated that *Olifantiella seblae* and *Labellicula lecohuiana* Majewska, Stefano & Van de Vijver (2017) could be conspecific in *Olifantiella gorandiana* complex (Riaux-Gobin et al., 2019). Likewise, *Achnanthes elongata* and *A*. *squaliformis* valves were 20.3–70 µm and 12.3–63.1 µm long respectively and showed high plasticity in this study. These two *Achnanthes* species were described with quite similar lengths to our samples in Majewska et al. (2017a); 15–75 µm for *A. elongata* and 11.5–45 µm for *A*. *squaliformis*).

### Biofilm composition

Our SEM observations of intact biofilms highlight that the biofilm is composed of microorganisms and mineral detritus along with micro-detritus from the carapace (Figs. 4 and 5). The formation of the biofilm seems to be a stochastic process, with the early colonisers (we observed diatoms) serving as a foundation for the subsequent deposition of organic and mineral detritus. A similar “messy” microstructure of the biofilm was also observed on the carapace of several species of sea turtles in Robinson et al. (2016). The biofilm observed on carapaces of olive ridley turtles from Costa Rica had a quite different spatial organisation (Majewska et al., 2015b). A relatively low diatom species number was reported (Majewska et al., 2015b), there was stable species composition with low inter-sample dissimilarities, and the epizoic microalgae were either partly immersed or entirely encapsulated within an exopolymeric coat. Here, the biofilm was formed by a massive occurrence of several diatom species with a dozen more taxa being sporadically observed. In addition, our observations on a clean carapace fragment revealed that among the micro-epibionts, diatoms might play a pioneering role as they attach with mucilage (Fuller et al., 2010) to the relatively smooth surface of the carapace.

The colonization of an existing surface by epibionts, organisms living attached to the body surface of a basibiont (host or substratum organism), constitutes one of the most substantial modifications of the basibiont’s body surface (Molino & Wetherbee, 2008; Wahl, 2008). Small epibionts, although generally ignored in the description of marine organisms, may have profound effects on the basibiont by causing a variety of either beneficial or detrimental effects. These effects should be taken into account when the ecology of the host is studied (Gillan & Cadee, 2000). Among the early settlers, microalgae play a crucial role in biofilm development and are able to settle on even the most fouling-resistant surfaces (Molino & Wetherbee, 2008).

### Biogeography

Our findings of biofilms composed of epizoic diatoms (e.g., *Achnanthes elongata*, *A*. *squaliformis, Olifantiella seblae, Tursiocola* sp. and *Tripterion* sp.) showed that the carapace of loggerhead sea turtles in the Mediterranean Sea was a suitable environment for diatom growth and further distribution. It was not possible to determine from which turtle population these epizoic diatoms originated, or from which substrata diatoms were introduced to the carapace (e.g., by grazing). There have not yet been sufficient comparable observations of sea turtle epizoic diatoms and the diatom flora from coasts or coral reefs of nesting grounds in general. However, the presence of epizoic diatom taxa of sea turtles from locations such as the Pacific Coast of Costa Rica, or the Caribbean and South American coasts (Majewska et al., 2015a; Majewska et al., 2015b; Riaux-Gobin et al., 2017a; Riaux-Gobin et al., 2017b; Riaux-Gobin et al., 2019), and the Mediterranean Sea might show that populations of basibionts meet somewhere in the oceans during their foraging migrations, as in the example of *Achnanthes squaliformis* or the similar species, *O*. *seblae*, *L*. *lecohuiana* both observed from the Atlantic and the Mediterranean sea turtles. In the Mediterranean *C. caretta* were tracked and it was found that turtles spent time foraging in the Eastern Mediterranean basin (Casale et al., 2012; Casale et al., 2013). It is possible that the Mediterranean loggerhead population and the Atlantic population could exchange diatom flora (Revelles et al., 2007). Genetic data have shown that the *C. caretta* populations from the west Atlantic coast spend time foraging with the population from the Eastern Mediterranean (Laurent et al., 1998; Carreras et al., 2006). Species distribution comparison of green sea turtles in Costa Rica and Iran showed a remarkable difference (Majewska et al., 2017b). Different characteristics of water column in Iranian coast of the Persian Gulf and Atlantic Ocean was found as a possible effect of diatom dispersal. Water chemistry and nutrients play a role in diatom community and their growth forms, where in the Persian Gulf species numbers were lower in the challenging environment. On the contrary, the Mediterranean loggerhead sea turtles comprised high biodiversity. However, tracking of these *C. caretta* is challenging and more detail is needed from the coasts of the Mediterranean for a comparison. But in the oligotrophic waters of the Eastern Mediterranean Sea diatom assemblage composition was significantly richer in species with epizoic diatoms present and characterized by high frequencies. Nonetheless, our study indicated that diatoms could adapt to the sea turtle’s microbiome and form a highly diversified facultative epibiont community. The dominant taxa in RA observed here were mostly raphid diatoms (in particular *Navicula* spp.). Raphid diatoms are generally among the earliest and most abundant primary colonizers of natural and artificial surfaces (Hoagland, Zlotsky & Peterson, 1986).

In general, the intensity of fouling pressure varies between season, latitude, depth, and local ecological factors; however, any permanently exposed, non-defended surface will eventually become fouled (Wahl, 1989). To determine whether seasonal and spatial variability is a relevant structuring factor, observations of the epibiont diatom community structure should be conducted involving more locations (e.g., different nesting grounds) in the Mediterranean, and over a more extended time period. Due to the fact that the nesting season only happens over a few months each year, there is little opportunity to study the seasonality of the diatom assemblages on the carapaces. However, as more and more turtles are tagged and followed with remote sensing (although it is still difficult to locate the tagged sea turtle as GPS trackers could be damaged or stop sending signals, and the turtle may not come back to ashore or to the same coasts for nesting in 3–4 years’ time), it could be possible and fruitful to repeat the analysis of the microbiome composition in terms of changes in the diatom assemblages over the time on the same sea turtle.

## Conclusions

Our study provides the first detailed information of the diatom assemblage from the *C. caretta* carapace. The results contain the diatom composition of sea turtle biofilm of the carapace and the specific taxa attached to carapace fragments. The most significant result from this study lies in the information about epibiont diatoms from turtles in this part of the Mediterranean Sea: there was great diatom diversity on the carapace, even if only some of them are considered epizoic forms. It seems that some of the epibionts are occurring on various host sea turtles e.g., *Achnanthes elongata* and have broad biogeography. This suggests that epibiont diatoms of the sea turtles have a broad range of ecological adaptation, even though some species were low in individuals, most species can survive in the biofilm, while the rare, typically epizoic, are well adapted.

The results of our study enhance the existing knowledge on the diatom-species composition and community structure of the microbiome of *C. caretta* carapaces in the Mediterranean, and will be a comparable dataset for *C. caretta* distributed in other geographic regions of ocean. Nesting sea turtles spend winters in the southeast Mediterranean (Isreal, Egypt, Tunisia), and as the diatom flora of these coasts has not been fully described, in general it is not possible to evaluate each individual sea turtle’s foraging area. Therefore, this study brings data for future comparison of nesting coasts from various locations, and contributes to sea turtle carapace flora of the Mediterranean *C. caretta* for further diatom investigations. Such knowledge would be useful for future investigations of sea turtles from different waters (East Atlantic coasts of Africa for the Mediterranean Sea turtle population).

##  Supplemental Information

10.7717/peerj.9406/supp-1 Supplemental Information 1 Tables S1-S6

10.7717/peerj.9406/supp-2 Supplemental Information 2 Raw Data A list of the observed species, SIMPER analysis of diatom contribution and dominant taxa habitat preferences.

## Additional Information and Declarations

### Field Study Permissions

The following information was supplied relating to field study approvals (i.e., approving body and any reference numbers): Field experiments were approved by DEKAMER- Sea Turtle Research, Rescue and Rehabilitation Centre (Ref. B.32.PAU.0.AG.00.00/005).

### Data Availability

The following information was supplied regarding data availability: The raw data are available in the Supplementary Files.
