# Supplementary material for: Biodiversity of carapace epibiont diatoms in loggerhead sea turtles (Caretta caretta Linnaeus 1758) in the Aegean Sea Turkish coast
Source: PeerJ. 2020 Jul 17;8:e9406. doi: 10.7717/peerj.9406 (PMC7370927; doi:10.7717/peerj.9406)
Supplement: Supplemental Information 1 [file peerj-08-9406-s001.pdf]

Table S1. A list of the taxa observed from the carapace of *C. caretta* between 2011-2014.

Cyclotella meneghiniana Kützing 1844

Pantocsekiella ocellata (Pantocsek) K.T .Kiss & Ács 2016

Melosira moniliformis (O. F. Müller) Agardh 1824

Hyalodiscus scoticus (Kützing) Grunow 1879

Paralia* cf. *sulcata (Ehrenberg) Cleve 1873

Lindavia balatonis (Pantocsek) Nakov *et al.* 2015

Odontella aurita Agardh 1832

Odontella rostrata (Hustedt) Simonsen 1987

Dimeregramma minus (Gregory) Ralfs 1861

Dimeregramma minus* var. *nanum (Gregory) Van Heurck 1896

***Dimeregramma* sp.**

Plagiogramma* cf. *pulchellum* var. *pygmaeum (Greville) H. Peragallo & M. Peragallo 1901

Brockmaniella brockmannii (Hustedt) Hasle, Stosch & Syvertsen 1983

Neohuttonia reichardtii (Grunow) Hustedt 1898

Plagiogramma tenuissimum Hustedt 1956

Anaulus balticus Simonsen 1959

Cymatosira belgica Grunow 1881

Cymatosira lorenziana Grunow 1862

Diatoma tenuis Agardh 1812

Fragilaria bronkei A. Witkowski, H. Lange-Bertalot & D. Metzeltin 2000

Fragilaria hyalina (Kützing) Grunow 1862

Hendeyella dubia (Grunow) Chunlian Li, Witkowski & Ashworth 2016

Hyalosynedra hyalina (Grunow) I. Álvarez-Blanco & S. Blanco 2014

Hyalosynedra* cf. *sublaevigata Blanco & Blanco 2014

***Hyalosynedra* sp.**

Neosynedra provincialis (Grunow) Williams & Round 1986

Opephora burchardtiae Witkowski, Metzeltin & Lange-Bertalot 1998

Opephora* cf. *guenter-grassii (Witkowski & Lange-Bertalot) Sabbe & Vyvermann 1995

Opephora minuta (Cleve-Euler) Witkowski, Lange-Bertalot & Metzeltin 2000

Opephora marina (Gregory) Petit 1888

Opephora mutabilis (Grunow) Sabbe & Vyverman 1995

Opephora pacifica (Grunow) Petit 1888

Tabularia fasciculata (Agardh) Williams & Round 1986

Tabularia tabulata (Agardh) Snoeijs 1992

Trachysphenia australis* var. *australis Petit 1877

Trachysphenia australis* var. *rostellata Hustedt 1955

Ulnaria ulna (Nitzsch) Compère 2001

Pseudostaurosiropsis geocollegarum (Witkowski) E. A. Morales 2002

***Staurosira* sp.**

Licmophora* cf. *hyalina (Kützing) Grunow 1867

Licmophora proboscidea Mereschkowsky 1902

***Licmophora* spp.**

Psammodiscus nitidus (Gregory) Round & Mann 1980

Delphineis australis (P. Petit) Watanabe *et al.* 2013

***Delphineis* sp.**

Diplomenora cocconeiformis (Schmidt) Blazé 1984

Meloneis mimallis I.Louvrou, D.B.Danielidis & A.Economou-Amilli 2013

***Meloneis* sp.**

***Thalassionema* sp.**

Grammatophora angulosa Ehrenberg 1840

Grammatophora angulosa* var. *mediterranea Grunow 1881

Grammatophora ocenica Ehrenberg 1840

Striatella unipunctata (Lyngbye) Agardh 1832

Achnanthes brevipes* var. *brevipes Agardh 1824

Achnanthes brevipes* var. *intermedia (Kützing) Cleve 1895

Achnanthes fimbriata (Grunow) Ross 1963

Achnanthes fogedii Håkansson 1978

Achnanthes elongata Majewska & Van de Vijver 2017

Achnanthes groenlandica* var. *phinneyi McIntire & Reimer 1974

Achnanthes parvula Kützing 1844

Achnanthes* cf. *pseudogroenlandica Hendey 1964

Achnanthes squaliformis Majewska & Van de Vijver 2017

Amphicocconeis disculoides (Hustedt) M. De Stefano & D. Marino 2002

***Amphicocconeis* sp.**

***Anorthoneis* sp.**

Cocconeiopsis* cf. *regularis (Hustedt) Witkowski, Lange-Bertalot & Metzeltin 2000

***Cocconeiopsis* spp.**

Cocconeis britannica Naegeli 1849

Cocconeis* cf. *distans Gregory 1855

Cocconeis clandestina A. Schmidt 1894

Cocconeis krammeri Lange-Bertalot & Metzeltin 1996

Cocconeis latecostata Hustedt 1955

Cocconeis peltoides Hustedt 1939

Cocconeis pinnata W. Gregory & Greville 1859

Cocconeis placentula Ehrenberg 1838

Cocconeis scutellum* var. *scutellum Ehrenberg 1838

***Cocconeis* spp.**

***Astartiella* sp.**

Karayevia amoena (Hustedt) Bukhtiyarova 2006

Karayevia submarina (Hustedt) Bukhtiyarova 2006

Planothidium* cf. *camppechianum (Hustedt) A. Witkowski, H. Lange-Bertalot & D. Metzeltin 2000

Planothidium delicatulun (Kützing) Round & Bukhtiyarova 1996

Planothidium depertidum (Giffen) A. Witkowski, H. Lange-Bertalot & D. Metzeltin 2000

Planothidium lanceolatum (Brébisson & Kützing) Lange-Bertalot 1999

Planothidium lilljeborgei (Grunow) Witkowski, Lange-Bertalot & Metzeltin 2000

***Chelonicola* spp.**

Rhoicosphenia abbreviata (Agardh) Lange-Bertalot 1980

Rhoicosphenia* cf. *adriatica Caput Mihalic & Levkov 2010

***Tripterion* spp.**

Dickieia* cf. *ulvaceae Berkeley 1844

***Dickieia* spp.**

Cymbella excisa Kützing 1844

Cymbella exica* var. *procera Krammer 2002

Cymbella exicisiformis Krammer 2002

Encyonema minutum (Hilse) D.G. Mann 1990

Navicymbula pusilla* var. *lata K. Krammer 2003

Lyrella abrupta (Gregory) Mann 1990

Lyrella amphoroides D.G. Mann 1997

Lyrella hennedyi (W.Smith) Stickle & D.G.Mann 1990

Lyrella lyra (Ehrenberg) Karajeva 1978

Navicula (Lyrella) lyra* var. *intermedia Peragallo & Peragallo 1897

Lyrella majuscula (Hustedt) Witkowski 1998

Navicula (Petroneis) humerosa* var. *arabica H. Peragallo & M. Peragallo 1898

Petroneis marina (Ralfs) Mann 1990

Mastogloia adriatica Voigt 1963

Mastogloia angusta Hustedt 1933

Mastogloia binotata (Grunow) Cleve 1895

Mastogloia biocellata (Grunow) Novarino & Muftah 1991

Mastogloia corsicana (Grunow) H. Peragallo & M. Peragallo 1897

Mastogloia crucicula (Grunow) Cleve 1895

Mastogloia crucicula* var. *alternans Zanon 1948

Mastogloia cuneata (Meister) Simonsen 1990

Mastogloia decipiens Hustedt 1933

Mastogloia decussata Grunow 1892

Mastogloia exilis Hustedt 1933

Mastogloia lanceolata Thwaites & W. Smith 1856

Mastogloia ovalis Schmidt 1893

Mastogloia* cf. *pumila Cleve 1895

Mastogloia pusilla* var. *pusilla Grunow 1878

***Mastogloia* spp.**

Tetramphora* cf. *lineolata (Ehrenberg) Mereschkowsky 1902

Berkeleya fennica Juhlin-Dannfelt 1882

***Climaconeis* spp.**

Parlibellus berkeleyi (Kützing) Cox 1988

Parlibellus* cf. *calvus Witkowski, Metzeltin & Lange-Bertalot 2000

Luticola nivalis (Ehrenberg) D.G. Mann 1990

Cistula lorenziana (Grunow) Cleve 1894

Halamphora acutiuscula (Kützing) Levkov 2009

Halamphora luciae (Cholknoy) Levkov 2009

Halamphora subholsatica (Krammer) Levkov 2009

Halamphora tenerrima (Aleem & Hustedt) Levkov 2009

Halamphora turgida (Gregory) Levkov 2009

Halamphora subangularis (Hustedt) Levkov 2009

Halamphora wisei (M.M. Salah) I. Álvarez-Blanco & S. Blanco 2014

***Halamphora* spp.**

Brachysira aponina Kützing 1836

Brachysira estonarium A. Witkowski, H. Lange-Bertalot & D. Metzeltin 2000

***Brachysira* sp.**

Olifantiella seblae Kaleli, Krzywda, Witkowski & Solak sp. nov.

Biremis lucens (Hustedt) Sabbe, Witkowski & Vyverman 1995

***Biremis* sp.**

Fallacia cassubiae Witkowski 1991

Fallacia florinae (Møller) Witkowski 1993

Fallacia forcipata (Greville) Stickle & Mann 1990

Fallacia* cf. *litoricola (Hustedt) D.G. Mann 1990

Fallacia nyella (Hustedt) D.G. Mann 1990

Fallacia oculiformis (Hustedt) D.G. Mann 1990

Fallacia schaeferae (Hustedt) D.G. Mann 1990

Fallacia* cf. *schoemaniana (Foged) Witkowski, Lange-Bertalot & Metzeltin 2000

Fallacia* cf. *spatiata (Østrup) Witkowski 2000

Fallacia subforcipata (Hustedt) D.G. Mann 1990

***Fallacia* spp.**

Diploneis aestuari Hustedt 1939

Diploneis bombus (Ehrenberg) Ehrenberg 1853

Diploneis caffra (Giffen) A. Witkowski, H. Lange-Bertalot & D. Metzeltin 2000

Diploneis coffaeiformis (Schmidt) Cleve 1894

Diploneis crabro (Ehrenberg) Ehrenberg 1854

Diploneis* cf. *mirabilis König 1959

Diploneis papula (A. Schmidt) Cleve 1894

Diploneis* cf. *parca (A. Schmidt) Boyer 1927

Diploneis incurvata* var. *dubia Hustedt 1933

Diploneis smithii (Brébisson) Cleve 1894

Diploneis subcincta (A. Schmidt) Cleve 1894

Diploneis suborbicularis* var. *constricta Hustedt 1937

Diploneis vacillans (A. Schmidt) Cleve 1894

Diploneis weissflogi (Schmidt) Cleve 1894

***Diploneis* spp.**

Fogedia finmarchia (Cleve & Grunow) Witkowski, Metzeltin & Lange-Bertalot 1997

Fogedia giffeniana (Foged) Witkowski, Lange-Bertalot, Metzeltin & Bafana 1997

***Fogedia* sp.**

Caloneis aemula (Grunow & A. Schmidt) Cleve 1894

Caloneis* cf. *excentrica (Grunow) Boyer 1927

Caloneis liber (W. Smith) Cleve 1894

Chamaepinnularia clamans (Hustedt) A. Witkowski, H. Lange-Bertalot & D. Metzeltin 2000

***Hippodonta* spp.**

Navicula arenaria* var. *rostellata Lange-Bertalot 1985

Navicula athenae Witkowski, Lange-Bertalot & Metzeltin 2000

Navicula* cf. *borowkae Witkowski, Lange-Bertalot & Metzeltin 2000

Navicula duerrenbergiana Hustedt 1934

Navicula flanatica Grunow 1860

Navicula normaloides Cholnoky 1968

Navicula* cf. *palpebralis Brébisson & W. Smith 1853

Navicula palpebralis* var. *angulosa (Gregory) Van Heurck 1885

Navicula palpebralis* cf. var. *minor Grunow 1880

Navicula palpebralis* cf. var. *undulata Peragallo & Peragallo 1897

Navicula pavillardii Hustedt 1939

Haslea spicula (Hickie) Bukhtiyarova 1995

Hippodonta subtilissima Lange-Bertalot, Metzeltin & Witkowski 1996

Navicula arenaria* var. *rostellata Lange-Bertalot 1985

Navicula athenae Witkowski, Lange-Bertalot & Metzeltin 2000

Navicula* cf. *borowkae Witkowski, Lange-Bertalot & Metzeltin 2000

Navicula duerrenbergiana Hustedt 1934

Navicula flanatica Grunow 1860

Navicula normaloides Cholnoky 1968

Navicula* cf. *palpebralis Brébisson & W. Smith 1853

Navicula palpebralis* var. *angulosa (Gregory) Van Heurck 1885

Navicula palpebralis* cf. var. *minor Grunow 1880

Navicula palpebralis* cf. var. *undulata Peragallo & Peragallo 1897

Navicula pavillardii Hustedt 1939

Navicula perminuta Grunow 1880

Navicula* cf. *ponticula Giffen 1970

Navicula ramosissima (C. Agardh) Cleve 1895

Navicula reichardtiana Lange-Bertalot 1989

Navicula salinicola Hustedt 1939

Navicula subagnita Proschkina-Lavrenko 1963

Navicula tripunctata (O.F. Müller) Bory 1822

Navicula* cf. *vekhovii Lange-Bertalot & Genkal 1999

Navicula viminoides Giffen 1975

***Navicula* spp.**

Seminavis robusta D.B. Danielidis & D.G. Mann 2002

Seminavis strigosa (Hustedt) Danielidis & Economou-Amilli 2003

Pinnunavis yarrensis (Grunow) Okuno 1975

Pinnularia claviculus (Gregory) Rabenhorst 1864

Gyrosigma balticum (Ehrenberg) Rabenhorst 1853

Gyrosigma eximium (Thwaites) Boyer 1927

Gyrosigma wansbeckii (Donkin) Cleve 1894

Pleurosigma elongatum W. Smith 1852

Pleurosigma strigosum W. Smith 1852

Toxonidea insignis Donkin 1858

Plagiotropis* cf. *lepidoptera (Gregory) Kuntze 1898

Amphora arcuata A. Schmidt 1875

Amphora bigibba* var. *interrupta (Grunow) Cleve 1895

Amphora cymbamphora Cholnoky 1960

Amphora* cf. *egregia Ehrenberg 1861

Amphora fluminensis Grunow 1863

Amphora graeffeana Hendey 1973

Amphora hyalina Kützing 1844

Amphora marina W. Smith 1857

Amphora montgomeryi A.H. Wachnicka & E.E. Gaiser 2007

Amphora pediculus (Kützing) Grunow 1875

Amphora polita Krasske 1939

Amphora proteus* var. *contigua Cleve 1895

Amphora proteus* var. *oculata H. Peragallo & M. Peragallo 1898

***Amphora* spp.**

Denticula subtilis Grunow 1862

***Denticula* sp.**

Nitzschia aequorea Hustedt 1939

Nitzschia amabilis H. Suzuki 2010

Nitzschia* cf. *plioveterana H. Lange-Bertalot 2000

Nitzschia* cf. *distans Gregory 1857

Nitzschia elegantula Grunow 1881

Nitzschia filiformis (W. Smith) Van Heurck 1896

Nitzschia frustulum (Kützing) Grunow 1880

Nitzschia grossestriata Hustedt 1955

Nitzschia inconspicua Grunow 1862

Nitzschia liebetruthii Rabenhorst 1864

Nitzschia lorenziana Grunow 1879

Nitzschia nana Grunow 1881

Nitzschia normannii Grunow 1881

Nitzschia pararostrata (Lange-Bertalot) Lange-Bertalot 2000

Nitzschia parvula W. Smith 1853

Nitzschia sigma (Kützing) W. Smith 1853

Nitzschia valdestriata Aleem & Hustedt 1951

Nitzschia volvendirostrata Ashworth, Dąbek & Witkowski 2016

***Nitzschia* spp.**

Psammodisctyon mediterraneum (Hustedt) Mann 1990

Psammodictyon panduriforme* var. *continua Snoeijs 1998

Psammodictyon panduriforme* var. *delicatulum (Grunow) Poulin 1990

Nitszchia (Psammodictyon) panduriforme* var. *minor Grunow

Psammodictyon roridum (Giffen) Mann 1990

Psammodictyon rudum (Cholnoky) D.G Mann 1990

***Tripterion* spp.**

Tryblionella apiculata Gregory 1857

Tryblionella compressa* var. *compressa (Bailey) Poulin 1990

Tryblionella compressa* var. *elongata (Grunow) Lange-Bertalot 1987

Tryblionella granulata (Grunow) D.G. Mann 1990

Tryblionella lanceola Grunow 1878

Tryblionella levidensis W. Smith 1856

Tryblionella navicularis (Brébisson) Ralfs 1861

Epithemia argus (Ehrenberg) Kützing 1844

Epithemia goeppertiana Hilse 1860

Epithemia smithii Carruthers 1864

Rhopalodia acuminata Krammer 1987

Rhopalodia musculus (Kützing) O. Müller 1900

Rhopalodia pacifica Krammer 1987

Entomoneis* cf. *paludosa (W. Smith) Reimer 1975

Petrodictyon gemma (Ehrenberg) Mann 1990

Surirella atomus Hustedt 1955

Surirella curvifacies Brun 1895

Surirella fastuosa (Ehrenberg) Ehrenberg 1843

Surirella striatula Turpin 1828

Table S2. SIMPER analysis of diatom taxa contributing (% cumulative = 90%) to similarities between epibiontic diatom assemblages from 2011.

**Taxa**	**Av.Abund**	**Av.Sim**	**Sim/SD**	**Contrib%**	**Cum.%**
Denticula subtilis Grunow	1.41	2.17	1.2	18.54	18.54
Fallacia subforcipata (Hustedt) Mann	1.53	1.7	0.58	14.52	33.05
Fallacia oculiformis (Hustedt) Mann	1.57	1.47	0.58	12.57	45.63
Halamphora tenerrima (Aleem & Hustedt) Levkov	2.28	1.35	0.58	11.57	57.2
Navicula cf. *palpebralis* Brébisson & Smith	1.37	1	0.58	8.59	65.79
Meloneis mimallis Louvrou et al.	0.89	1	0.58	8.57	74.36
Achnanthes fogedii Håkansson	0.91	0.96	0.58	8.18	82.55
Biremis lucens (Hustedt) Sabbe et al.	1.06	0.93	0.58	7.95	90.5

Table S3. **SIMPER analysis of diatom taxa contributing (% cumulative = 91%) to similarities between epibiontic diatom assemblages from 2012.**

**Taxa**	**Av.Abund**	**Av.Sim**	**Sim/SD**	**Contrib%**	**Cum.%**
Nitzschia frustulum (Kützing) Grunow	4.29	5.41	1.06	20.13	20.13
Navicula perminuta Grunow	2.17	2.66	1.12	9.90	30.03
Nitzschia liebetruthii Rabenhorst	1.71	2.44	1.09	9.09	39.12
Tripterion sp.2	1.99	2.13	0.83	7.93	47.05
Halamphora tenerrima (Aleem & Hustedt) Levkov	1.66	1.60	0.62	5.97	53.02
Delphineis australis (Petit) Watanabe et al.	1.37	1.49	0.94	5.54	58.56
Navicula normaloides Cholnoky	0.93	1.02	1.12	3.80	62.36
Halamphora luciae (Cholnoky) Levkov	1.47	0.94	0.54	3.50	65.86
Cocconeis placentula Ehrenberg	0.62	0.81	1.15	3.03	68.89
Karayevia submarina (Hustedt) Bukhtiyarova	0.55	0.81	1.15	3.03	71.92
Tabularia fasciculata (Agardh) Williams & Round	0.59	0.62	0.62	2.30	74.22
Nitzschia sp.20	0.62	0.55	0.58	2.06	76.28
Nitzschia sp.19	0.52	0.53	0.61	1.97	78.25
Amphora marina Smith	0.91	0.50	0.59	1.85	80.10
Tryblionella granulata (Grunow) Mann	0.95	0.49	0.60	1.84	81.93
Diploneis bombus (Ehrenberg) Ehrenberg	0.54	0.44	0.61	1.65	83.59
Cocconeis sp.19	0.44	0.44	0.62	1.65	85.24
Brachysira estonarium Witkowski et al.	0.71	0.44	0.62	1.64	86.88
Tryblionella lanceola Grunow	0.42	0.40	0.62	1.50	88.38
Melosira moniliformis (Müller) Agardh	0.58	0.39	0.62	1.45	89.82
Tryblionella apiculata Gregory	0.71	0.32	0.32	1.20	91.02

Table S4. SIMPER analysis of diatom taxa contributing (% cumulative = 90%) to similarities between epibiontic diatom assemblages from 2013.

**Taxa**	**Av.Abund**	**Av.Sim**	**Sim/SD**	**Contrib%**	**Cum.%**
Delphineis australis (Petit) Watanabe et al.	2.25	2.57	0.75	17.88	17.88
Grammatophora angulosa Ehrenberg	1.61	1.53	0.55	10.64	28.52
Navicula perminuta Grunow	1.51	1.32	0.54	9.18	37.69
Meloneis mimallis Louvrou et al.	1.14	1.06	0.57	7.39	45.09
Nitzschia frustulum (Kützing) Grunow	1.31	0.84	0.46	5.82	50.91
Delphineis sp.1	0.97	0.7	0.49	4.85	55.76
Diplomenora cocconeiformis (Schmidt) Blazé	0.84	0.56	0.4	3.9	59.66
Achnanthes elongata Majewska & Van de Vijver	1.27	0.53	0.17	3.68	63.33
Tryblionella granulata (Grunow) Mann	0.87	0.38	0.42	2.63	65.96
Nitzschia liebetruthii Rabenhorst	0.76	0.35	0.33	2.41	68.37
Cocconeis scutellum Ehrenberg	0.66	0.28	0.29	1.95	70.32
Olifantiella sp.1	0.52	0.27	0.39	1.89	72.21
Achnanthes squaliformis Majewska & Van de Vijver	0.55	0.23	0.2	1.59	73.8
Navicula sp.54	0.66	0.22	0.34	1.55	75.35
Nitzschia sp.20	0.56	0.21	0.24	1.44	76.8
Psammodictyon rudum (Cholnoky) Mann	0.7	0.2	0.22	1.41	78.21
Cocconeis placentula Ehrenberg	0.43	0.18	0.32	1.27	79.48
Navicula normaloides Cholnoky	0.63	0.18	0.26	1.24	80.72
Tripterion sp.2	0.76	0.17	0.15	1.22	81.93
Halamphora tenerrima (Aleem & Hustedt) Levkov	0.38	0.13	0.21	0.92	82.86
Melosira moniliformis (Müller) Agardh	0.44	0.12	0.28	0.86	83.72
Diploneis bombus (Ehrenberg) Ehrenberg	0.33	0.11	0.25	0.79	84.52
Tryblionella lanceola Grunow	0.33	0.11	0.29	0.75	85.27
Paralia sulcata (Ehrenberg) Cleve	0.28	0.11	0.28	0.75	86.02
Amphora polita Krasske	0.3	0.11	0.25	0.74	86.76
Navicula sp.55	0.42	0.1	0.23	0.7	87.46
Achnanthes pseudogroenlandica Hendey	0.31	0.09	0.18	0.63	88.1
Cymatosira lorenziana Grunow	0.32	0.09	0.22	0.61	88.7
Amphora marina Smith	0.26	0.06	0.22	0.45	89.15
Mastogloia pusilla var. *pusilla* Grunow	0.24	0.06	0.23	0.42	89.57
Diploneis coffaeiformis (Schmidt) Cleve	0.25	0.06	0.17	0.41	89.98
Cocconeis sp.8	0.3	0.06	0.13	0.41	90.39

Table S5. SIMPER analysis of diatom taxa contributing (% cumulative = 90%) to similarities between epibiontic diatom assemblages from 2014.

**Taxa**	**Av.Abund**	**Av.Sim**	**Sim/SD**	**Contrib%**	**Cum.%**
Navicula perminuta Grunow	3.03	3.69	6.51	9.73	9.73
Nitzschia frustulum (Kützing) Grunow	3.13	2.77	2.31	7.31	17.04
Cocconeis placentula Ehrenberg	2.17	2.43	2.03	6.41	23.45
Navicula sp.54	2.03	2.27	1.67	5.97	29.42
Navicula sp.55	1.92	2.01	2.41	5.29	34.71
Nitzschia liebetruthii Rabenhorst	1.48	1.68	2.5	4.41	39.12
Melosira moniliformis (Müller) Agardh	1.87	1.64	1.33	4.33	43.45
Tryblionella granulata (Grunow) Mann	1.31	1.51	2.46	3.98	47.43
Seminavis strigosa (Hustedt) Danielidis & Economou-Amilli	1.32	1.21	1.49	3.2	50.63
Navicula sp.52	1.35	1.05	1.67	2.77	53.4
Tryblionella apiculata Gregory	0.91	0.91	1.76	2.4	55.8
Delphineis australis (Petit) Watanabe et al.	1.65	0.85	0.72	2.24	58.04
Olifantiella sp.1	1.15	0.83	1.02	2.18	60.23
Tabularia fasciculata (Agardh) Williams & Round	1.54	0.78	0.98	2.04	62.27
Diploneis cf. *mirabilis* König	0.89	0.72	0.93	1.91	64.18
Karayevia submarina (Hustedt) Bukhtiyarova	0.81	0.72	1.04	1.9	66.07
Grammatophora angulosa Ehrenberg	0.85	0.71	1.06	1.87	67.94
Achnanthes elongata Majewska & Van de Vijver	0.88	0.65	0.96	1.7	69.64
Nitzschia sp.20	0.97	0.58	1.06	1.53	71.17
Cocconeis scutellum Ehrenberg	0.75	0.55	0.87	1.46	72.63
Paralia sulcata (Ehrenberg) Cleve	0.69	0.54	1.15	1.43	74.06
Delphineis sp.1	0.87	0.54	1.05	1.43	75.49
Psammodictyon panduriforme var. *continua* Snoeijs	0.74	0.51	0.86	1.34	76.82
Navicula normaloides Cholnoky	0.7	0.48	0.83	1.26	78.09
Diploneis bombus Ehrenberg (Ehrenberg)	0.51	0.47	1.1	1.24	79.32
Tripterion sp.2	0.76	0.47	0.78	1.23	80.56
Navicula pavillardii Hustedt	0.68	0.46	1.02	1.22	81.77
Amphora marina Smith	0.67	0.43	0.99	1.13	82.9
Psammodictyon rudum (Cholnoky) Mann	0.56	0.36	0.61	0.95	83.84
Navicula salinicola Hustedt	0.41	0.35	1.07	0.93	84.77
Rhopalodia pacifica Krammer	0.48	0.29	0.62	0.76	85.53
Tryblionella lanceola Grunow	0.67	0.27	0.48	0.71	86.25
Meloneis mimallis Louvrou et al.	0.79	0.27	0.46	0.71	86.96
Tryblionella compressa var. *elongata* (Grunow) Lange-Bertalot	0.75	0.27	0.46	0.71	87.67
Amphora sp.10	0.43	0.26	0.61	0.68	88.35
Cymbella excisa Kützing	0.42	0.24	0.61	0.64	88.98
Halamphora acutiuscula (Kützing) Levkov	0.36	0.23	0.6	0.61	89.6
Diploneis coffaeiformis (Schmidt) Cleve	0.42	0.2	0.62	0.53	90.13

Table S6. The ecological distribution of dominant taxa (1- Guiry & Guiry, 2019; 2- Kociolek et al. 2019, 3- Witkowski et al., 2000; M- Marine, B- Brackish & F- Freshwater).

Taxa	1	2	3	Taxa	1	2	3
***Achnanthes elongata* Majewska & Van de Vijver**	**M**	**-**	**-**	*Navicula perminuta* Grunow	**M,B**	**M,F**	**B**
***Delphineis australis* (Petit) Watanabe et al.**	**M**	**M**	**M**	*Navicula viminoides* Giffen	**M,F**	**M,B,F**	**M,B**
***Encyonema minutum* (Hilse) D.G.Mann**	**F**	**M,F**	**-**	*Neosynedra provincialis* (Grunow) Williams & Round	**M**	**F**	**M**
***Fallacia cassubiae* Witkowski**	**-**	**-**	**B**	*Nitzschia frustulum* (Kützing) Grunow	**M,F**	**M,F**	**M,B**
***Fallacia florinae* (Møller) Witkowski**	**M**	**M**	**M,B**	*Nitzschia volvendirostrata* Ashworth et al.	**M**	**M**	**-**
***Grammatophora angulosa* Ehrenberg**	**M**	**M,F**	**M**	*Olifantiella seblae* Kaleli et al.	**-**	**-**	**-**
***Halamphora tenerrima* (Aleem & Husted) Levkov**	**M,B**	**M**	**M**	*Psammodictyon rudum* (Cholnoky) Mann	**M**	**M**	**M**
***Mastogloia crucicula* var. *alternans* Zanon**	**-**	**-**	**M**	*Rhoicosphenia abbreviata* (Agardh) LangeBertalot	**F**	**M**	**F,B**
***Navicula palpebralis* var. *angulosa* (Gregory) Van Heurck**	**F**	**M,B**	**M**				
